# The role of hypoxia on prostate cancer progression and metastasis

**DOI:** 10.1007/s11033-023-08251-5

**Published:** 2023-02-14

**Authors:** Osama A. A. Mohamed, Heba S. Tesen, Marwa Hany, Aya Sherif, Maya Magdy Abdelwahab, Muhammed H. Elnaggar

**Affiliations:** 1 Biotechnology Department, Faculty of Science, Mansoura University, Dakahlia, Egypt; 2 Faculty of Medicine, Ain Shams University, Cairo, Egypt; 3 Biotechnology Department, Faculty of Science, Cairo University, Giza, Egypt; 4 Chemistry & Microbiology Department, Faculty of Science, Cairo University, Giza, Egypt; 5 Faculty of Medicine, Helwan University, Cairo, Egypt; 6 Biochemistry Department, Faculty of Science, Ain Shams University, Cairo, Egypt; 7 Biomedical Research Department, Tetraploid Team, Cairo, Egypt

**Keywords:** Hypoxia, Prostate cancer, Metastasis, HIF, EMT

## Abstract

Prostate cancer is the second most common cancer diagnosed in men and the fifth-leading cause of cancer death in men worldwide. Like any solid tumor, the hypoxic microenvironment of prostatic cancer drives hypoxia-inducible factors (HIFs) to mediate cell adaptions to hypoxic conditions. HIFs direct different signaling pathways such as PI3K/Akt/mTOR, NOX, and Wnt/β-Catenin to tumor progression depending on the degree of hypoxia. HIFs regulate cytoskeleton protein expression, promoting epithelial-mesenchymal transition (EMT), which occurs when cancer cells lose cell-to-cell adhesions and start invasion and metastasis. Through activating pathways, the hypoxic microenvironment maintains the self-renewal, potency, and anti-apoptotic function of prostate cancer cells and induces tumor metastasis and transformation. These pathways could serve as a potential target for prostate cancer therapy. HIFs increase the expression of androgen receptors on cancer cells maintaining the growth and survival of prostate cancer and the development of its castration resistance. In this review, we elaborate on the role of hypoxia in prostatic cancer pathogenesis and different hypoxia-induced mechanisms.

**Supplementary Information:**

The online version contains supplementary material available at 10.1007/s11033-023-08251-5.

## Introduction

Prostate cancer is the second most commonly diagnosed malignancy in men worldwide, with about 1.3 million cases diagnosed with prostate cancer in 2018 [1, 2]. Prostate cancer spreads locally to the pelvic organs or metastasizes distantly in midline bones, liver, and lung causing fatal complications [3, 4]. Cancer metastasis reduces the survival rate to one-third, and metastasis to bone and vertebral column causes sensory and motor neurological disorders. In many solid tumors, cancer cells are exposed to hypoxia due to insufficient angiogenesis and cancer progression [5, 6]. Hypoxia occurs in the early stages of prostate cancer due to the accumulation of acidic metabolites or reactive oxygen species (ROS) [7]. Hypoxia-inducible factors (HIFs) accumulate inside cells to adapt to hypoxic conditions and manage the tumor microenvironment [8]. HIFs affect other microenvironment components such as blood vessels, lymphatic vessels, and immune cells and further affect extracellular matrix remodeling [9].

Hypoxia-inducible factors are degraded by adding a hydroxyl group to proline residue by oxygen-dependent prolyl hydroxylation enzyme (PHDs), hence, many signaling pathways are activated to stabilize HIFs [8]. Hypoxia drives epithelial-mesenchymal transition (EMT) and tumor metastasis. Under the influence of HIF-1, prostate cancer cells lose the epithelial cell marker E- cadherin and gain mesenchymal cell marker N–cadherin.

Little information is available about how hypoxia is initiated in prostate cancer cells and how it contributes to cancer progression, stemness, and metastasis. This review discusses the hypoxic microenvironment in prostate cancer and the activated signaling pathways. Additionally, we interpret how hypoxia contributes to the self-renewal and anti-apoptotic function of prostate cancer cells.

## Hypoxia and prostate cancer pathogenesis

### Hypoxic tumor microenvironment

The stromal compartments (tumor microenvironment components) are non-malignant cells such as blood vessels, lymphatic vessels, fibroblasts, and extracellular matrix (ECM). The interaction between the stromal compartments and the cancer cells plays a vital role in tumor progression and growth [10]. A hypoxic tumor is a condition in which both cancer and stromal cells have minimal oxygen concentration. HIFs play a major role in this condition. HIF-dependent signaling enhances the interaction between cancer and stromal cells, facilitating cancer progression [11]. Also, HIFs transcriptional activities cause the release of some signaling molecules from cancer and stromal cells leading to an increase in the acidification and the exhaustion of glucose, pyruvate, and lactate facilitating cancer development [9].

### Cancer-associated fibroblasts (CAFs)

Cancer-associated fibroblasts (CAFs) are an important component of stromal cells that form 80% of the tumor and have many roles in tumor regulation and shaping [12]. Hypoxia induces the cancer-associated myofibroblasts to release cytokines such as CXCL13 which are an inducer of cancer progression [13]. Hypoxic cancer cells produce paracrine signaling molecules such as the TGF-β family and CTGF where HIF-1 regulates these molecules to convert the progenitor cells into CAFs [14]. This type of CAFs has high levels of HIF-1α thus, it undergoes an elevated level of aerobic glycolysis [15]. Then, the surrounding cancer cells deplete lactate resulting in a high rate of glycolysis and enhancing tumor progression.

### Extracellular matrix

The extracellular matrix (ECM) consists of fibrillar proteins such as collagens (90% of ECM) and proteoglycans. EMC coordinates tissue homeostasis, inflammation, cell adhesion, and migration [16, 17]. CAFs in prostate cancer have high production of EMC proteins (fibrosis) and remodeling enzymes which induce angiogenesis, epithelial cell growth, and migration [18]. HIFs induce the expression of collagen-modifying enzymes such as prolyl-4-hydroxylase alpha-subunits (P4HA1 and P4HA2) and procollagenlysyl-hydroxylases (PLOD1 and PLOD2), which are essential for collagen synthesis and collagen fibrils synthesis [19]. Furthermore, HIF-1 stimulates the production of lysyl-oxidase LOX [20], LOX12 [21], and LOX4 [22], which are essential for the deamination of collagen fibrils on lysine and hydroxylysine residues. This deamination is necessary for the consistency of collagen fiber and fibril cross-linking [23].

### Blood vessels

Excessive formation of vessels leads to the progress of the tumor from hyperplasia to neoplasia [24]. The blood vessels consist mainly of endothelial cells and pericytes. Endothelial cells lining blood vessels and their branches expand away from the main vessels [25]. These vessels deliver oxygen to tissues, remove waste products and produce growth factors with autocrine effects such as VEGF (vascular endothelial growth factor). Angiogenesis is the development of new vascular vessels from pre-existing blood vessels [26]. VEGF is the most important angiogenesis activator related to the metastatic capability that enhances the hyperpermeability of vessels, endothelial cell proliferation, and endothelial cell migration. Hypoxia has important mechanisms for increasing VEGF expressions such as the binding of HIF-1 to FMS-like tyrosine kinase-1 and fetal liver kinase 1. Also, hypoxia increases the VEGF expression by regulating HIF-1 PI3/AKT/FRAP pathway [27].

### Immune cells

Since the immune system plays a crucial role in tumors defense, malignant tumors require immunosuppressive cells including CD4 + FoxP3 + regulatory T cells, anti-inflammatory M2 macrophages, and myeloid-derived suppressor cells (MDSCs), as well as their factors such as prostaglandin E2 (PGE2), for spreading and resistance to immune cell [28]. Also, tumor cells suppress immune cells by enhancing the expression of immune checkpoints such as PDL-1. In hypoxic conditions, HIF-1 enhances PDL-1 expression by binding the HIF‐1α binding site HRE4 in the PD‐L1 proximal promoter. PDL-1ligand interacts with the PD-1 receptor on T cells to inactivate T cells and inhibit their function [29]. HIF-1 affects miR-224, which regulates the signals between NK cells and their receptors (NKp46/NCR1 with a mouse ortholog) and reduces the ability of NK cells to kill cancer cells [30].

### Hypoxia signaling pathways

#### HIF Signaling pathway

HIF is a heterodimeric protein consisting of one constitutively expressed HIF-β subunit (which is known as the nuclear translator of the aryl hydrocarbon receptor) and one of three oxygen-dependent HIF-α subunits (HIF-1α, HIF-2α, or HIF-3α). The molecular weight of the α-subunit is 120–130 kDa; the β-subunit is 91–94 kDa. Both α and β subunits belong to the basic family of transcription factors, which include bHLH (helix—loop—helix) and PAS (PER-ARNT-SIM) domains. The bHLH domain is responsible for binding the transcription factor HIF-1 to the HRE DNA region (hormone response element), while the PAS domain is involved in the dimerization of HIF-1 [31].

HIF-1α is activated in physiologically important places of regulation of oxygen metabolism pathways, providing quick and adequate responses to hypoxic stress by acting on target genes that regulate the process of angiogenesis, vasomotor control, energy metabolism, erythropoiesis, and apoptosis. Along with this, HIF-1 is involved in cell death/survival processes, glucose metabolism, cell migration and invasion, cell microenvironment remodeling, carcinogenesis, metastasis, and many others [32]. HIF-2α (also known as EPAS1), which shares 48% amino acid identity with HIF-1α, is also induced by hypoxia, dimerizes with HIF-1β, and activates the transcription of target genes, some of which are common with HIF-1α [31]. A third HIF protein, HIF-3α, was discovered in 1998. A variant of HIF-3α splicing is IPAS protein (PAS domain inhibitor). IPAS has a negative regulatory function and acts as a factor in the downregulation of HIF1α and HIF2α. It binds to the N-terminal region of HIF-1α and prevents its binding to DNA [33].

**Fig.1:**
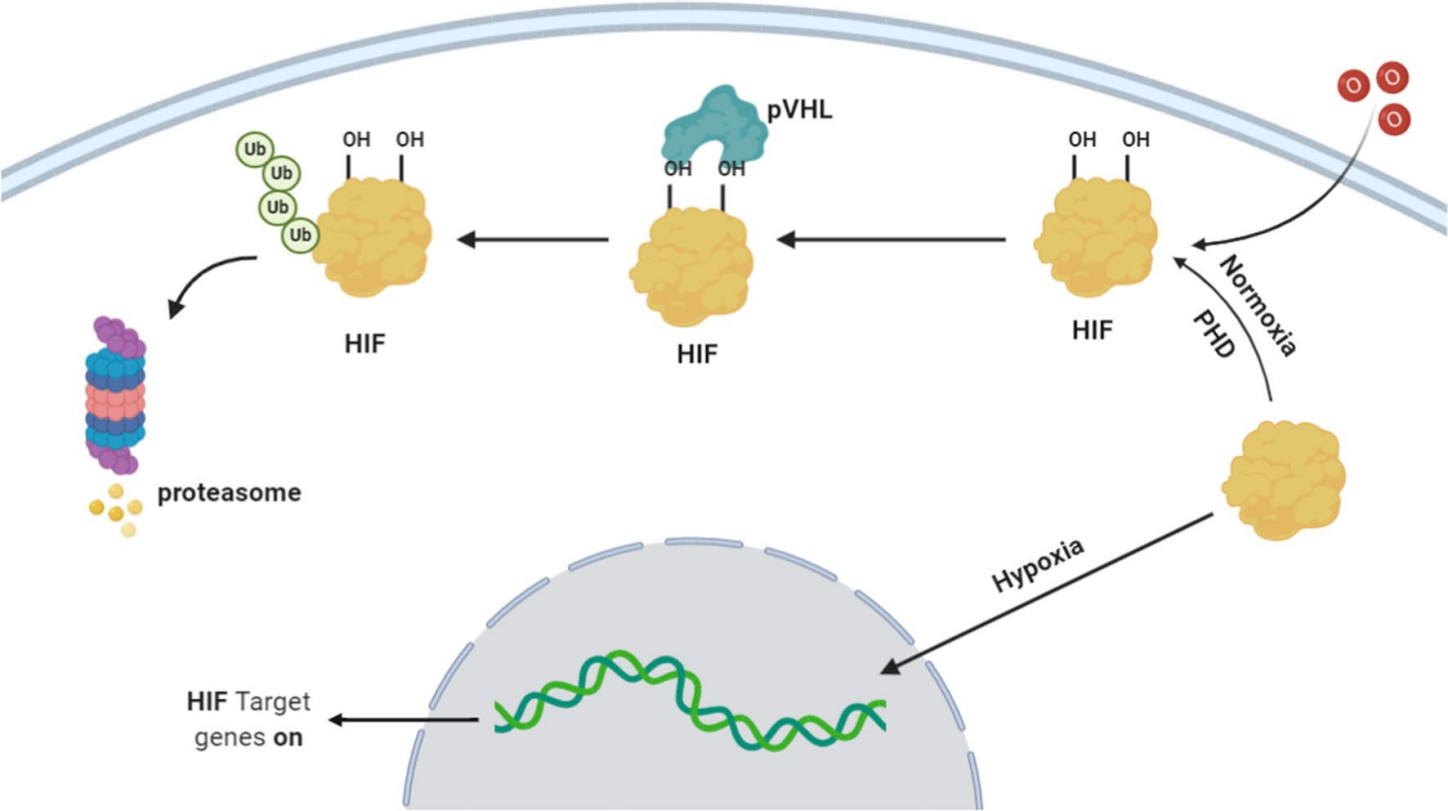
HIF Signaling Pathway: Under hypoxic conditions, the decrease in oxygen inhibits HIF-1α hydroxylation and allows its translocation to the nucleus, where it binds to HIF-1β to form an active heterodimer complex as well as interacts with CBP/p300 transcription coactivators and ultimately to the activation of transcription of regulated HIF-1 genes including angiogenesis, glucose transporters, proliferation, metastasis, stemness, and drug resistance genes

Under normoxic conditions, as shown in Fig. 1, HIF 1α subunits are constantly present in the cell but are unstable and rapidly undergo degradation, controlled by hydroxylation of prolyl and asparagine. The presence of oxygen triggers the hydroxylation of the HIF proline residue [34]. This hydroxylation is catalyzed by the family of intracellular proline hydrolases (PHDs). PHDs are 2-oxoglutarate-dependent dioxygenases (2-OG). For the manifestation of their catalytic activity, Fe2 + ions are required. In the human body, there are three different PHDs of HIF-1α: PHD-1 is located exclusively in the cell nucleus, PHD-2 is predominantly localized in the cytoplasm, and PHD3 is found both in the nucleus and in the cytoplasm [35]. Oxygen interacts with Fe2 + , which is part of the active center of PHDs, with one oxygen atom participating in the hydroxylation of proline while the other in the conversion of 2-oxoglutarate to succinate and CO_2_. For this reaction to occur, ascorbate is also necessary, which prevents the spontaneous oxidation of Fe2 +  [36]. Immediately after hydroxylation at proline residues, HIF-1α binds to the von Hippel-Lindau protein (VHL), which serves as a substrate for ubiquitin protein ligase E3. This leads to the polyubiquitination of HIF-1α and its further degradation with the participation of the 26S proteasome [34]. The presence of oxygen also causes the hydroxylation of the aspartic residue C of the terminal transactivation domain (C-TAD) of HIF 1α, blocking the interaction with the p300/CBP transcription activator [37]. This process is regulated by a specific asparagine hydroxylase called FIH-1 (Factor inhibiting HIF-1). As a result, in the presence of oxygen, the active enzymes PHD and FIH inactivate HIF, thereby blocking HIF-mediated gene transcription [38].

Under hypoxic conditions, the decrease in oxygen level inhibits HIF-1α hydroxylation and allows for its translocation to the nucleus, where it binds to HIF-1β to form an active heterodimer complex as well as interacts with CBP/p300 transcription coactivators and ultimately to the activation of transcription of regulated HIF-1 genes including angiogenesis, glucose transporter, proliferation, metastasis, stemness, and drug resistance genes [39].

#### PI3K/Akt/mTOR signaling pathway

**Fig.2:**
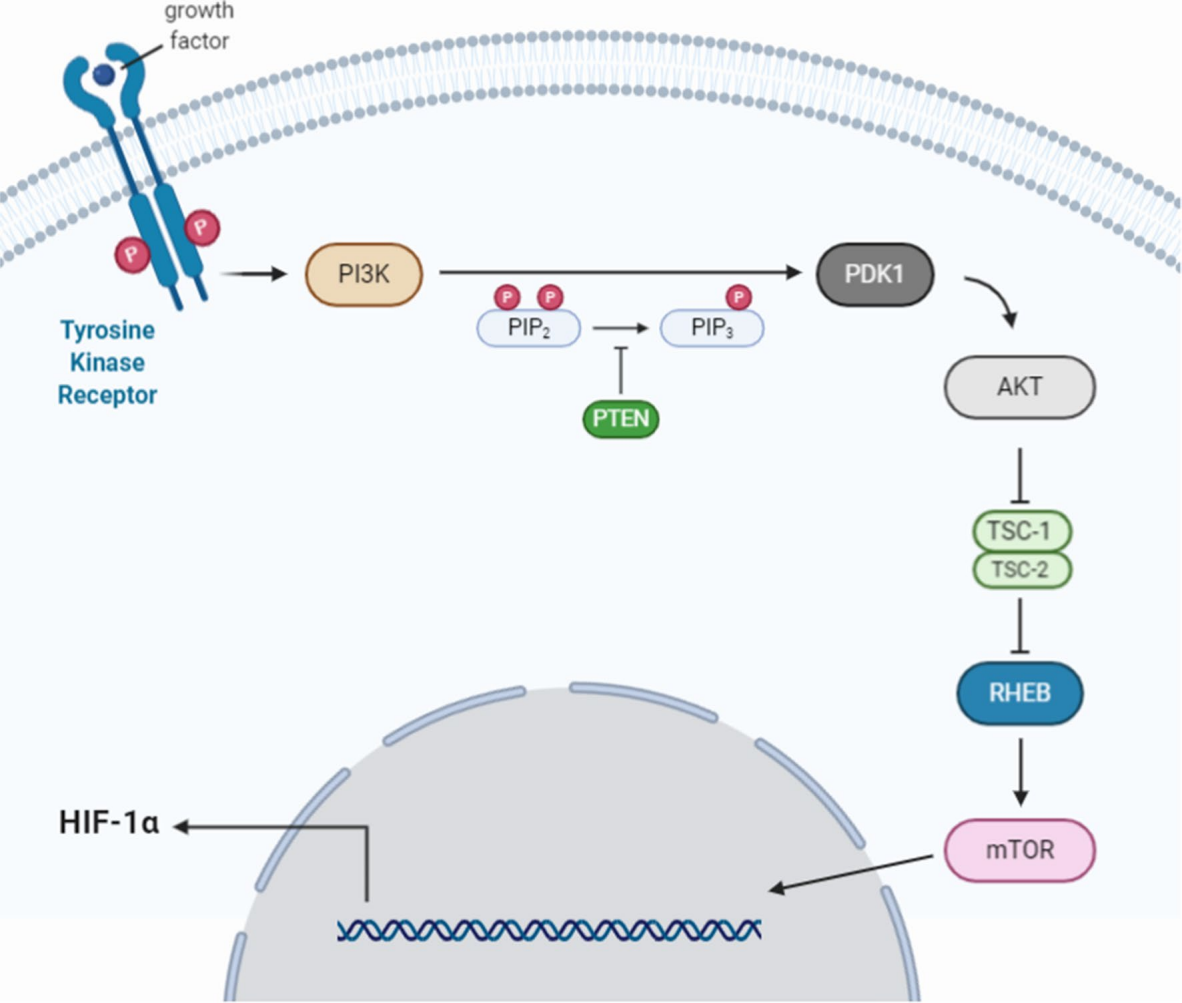
PI3K/Akt/mTOR Signaling pathway: When receptor kinases activate PI3K, phosphorylates phosphoinositol to 3-phosphoinositide, which, in turn, transactivates several enzymes, including protein kinase B (PKB). Akt activation occurs indirectly through the phosphorylation of intermediate protein kinases (for example, PDK-1), the substrates of which are protein kinases (Akt, PKC). Activated Akt inhibits the function of the TSC-2 protein complex, which normally suppresses mTOR-induced activation of the transcriptional and translational regulatory processes

The PI3K/Akt/mTOR signaling pathway, shown in Fig. 2, is an intracellular signaling pathway important in regulating the cell cycle. Therefore, it is directly related to cell growth, proliferation, and apoptosis.PI3K (phosphatidylinositol-3-kinase) is a membrane-bound kinase that, when activated by receptor kinases, phosphorylates the 3'-hydroxyl group of the inositol ring of phosphatidylinositol (PI), generating several phosphatidylinositol isoforms. This is achieved via a series of reactions catalyzed by proteins, including PI3K and its metabolite protein, phosphoinositide-dependent kinase 1 (PDK1), and by the protein Akt. These isomers include phosphatidylinositol-3,4,5-trisphosphate (PIP3), phosphatidylinositol-3,4-bisphosphate (PIP2), and phosphatidylinositol-4,5-bisphosphate (PIP). Also, these phosphatidylinositol isoforms serve as important second messenger molecules that regulate a wide range of cellular processes. Along with this, PI3K acts as a second messenger to recruit Akt and PDK1. PDK1 can partially activate Akt through the phosphorylation of the Thr308 site, while the phosphorylation of the Ser473 site by mTORC2 can fully activate Akt, and fully activated Akt can activate a large number of signals that regulate growth, proliferation, metabolism, or apoptosis through phosphorylation molecule [40]. Akt and other signaling molecules regulate the TSC complex consisting of tuberous sclerosis complex subunit 1 (TSC1) and TSC2 [41]. In addition, Akt induces phosphorylation of the proline-rich Akt substrate of 40 kDa (PRAS40), which in turn degrades its inhibitory interaction with mTOR [42], thereby fully activating the mTOR kinase. At the same time, the activation of AKT leads to the phosphorylation of glycogen synthase kinase 3 (GSK3), leading to its inhibition. The GSK3 has been linked to the regulation of an assembly of transcription factors, including β-catenin, which is a core component of the Wnt/β-catenin signaling pathway as it activates the transcription of crucial target genes responsible for cellular proliferation and differentiation. Therefore, Akt is a positive regulator of the Wnt/β-catenin signaling pathway through the inhibition of GSK3 [43].

Directing the PI3K/Akt/mTOR signaling pathway seems to be a promising antitumor therapy strategy since the kinases involved in this pathway are constantly activated for several types of cancer (including PCa cells). This activation is associated with an unfavorable prognosis of the course and resistance of the tumors to chemotherapy [44]. Activation of the PI3K/Akt/mTOR pathway stimulates the expression of hypoxia-induced factor 1α (HIF-1α), which is an essential regulator of angiogenesis [45, 46]. HIF1α expression is enhanced under hypoxic conditions and stimulates tumor cells to secrete vascular endothelial growth factor (VEGF) and platelet-derived growth factor (PDGF) [47]. A previous report showed that inhibiting the PI3K/Akt/mTOR pathway with LY294002 in PCa cells could significantly inhibit the expression of HIF-1α [48].

Many studies investigate how the PI3K/Akt/mTOR pathway is related to HIF-1α expression under hypoxia. A study by Hudson et al. showed that mTOR could regulate the expression of HIF-1α as it can affect the oxygen-dependent degradation domain in HIF-1α [49]. Another study by Joshi et al. showed that activation of PI3K/Akt signaling or PTEN inactivation under hypoxia causes resistance of HIF-1α to degradation [50]. PTEN phosphatase catalyzes the removal of the phosphate group at the 3D position of the inositol ring of PI3K, inhibiting signal transmission along the PI3K/Akt/mTOR signaling pathway [51]. A study by Movafagh et al. also showed that under hypoxia, the reactive oxygen species (ROS) could activate the PI3K/Akt/mTOR signaling pathway, which then increases the expression of HIF-1α [52]. Briefly, the PI3K/Akt/mTOR signaling pathway has a significant role in the progression of PCa. It could serve as a potential target for prostate cancer therapy under the hypoxic microenvironment.

#### NOX signaling pathway

The NOX system is an enzyme complex consisting of several protein subunits. Two of them: gp91phox (β-subunit) and p22phox (α-subunit), are localized on the membrane and form flavocytochrome b558. The remaining four subunits: p47-phox, p67-phox, p40- phox, GTPase Rac1 or Rac2 are cytoplasmic [53]. The p40-phox subunit is responsible for the resting state of the NOX system. Upon activation, it is separated from p47 and p67, which simultaneously acquire the ability to conjugate with membrane subunits [54]. The NOX system carries out transmembrane electron transfer to O_2_ and forms a superoxide anion radical. The reactive oxygen species (ROS) formed by NOX regulate the activity of neutrophils and the degradation of membrane phospholipids by activating various forms of phospholipase A2 (PLA2): cytosolic cPLA2 and secretory sPLA2, and also participate as secondary messengers in the transmission of signals to the cell [54]. NOX-mediated ROS production activates many oncogenes such as receptor tyrosine kinases (*e.g*., EGFR), small GTPases (*e.g*., Ras), serine/threonine kinases (*e.g*., Raf and Akt), cytoplasmic tyrosine kinases (*e.g*., Src), as well as lipid kinases (*e.g*., PI3Ks). Moreover, it inactivates many tumor suppressor genes such as PTEN, p53, and TSC2, thereby deemed pivotal in the transformation and maintenance of resistant and malignant phenotypes in cancer cells [55, 56]. Several studies found that the NOX system is associated with some tumor growth and progression, such as in breast and prostate cancer. A study by Ramesh et al. reported that in breast cancer, ROS activates many signaling pathways involved in cell proliferation and survival, leading to breast cancer progression [57]. Another study by Lim et al. showed that the increase in human prostate tumors tumorigenicity is associated with overexpression of NOX1 and ROS formation [58].

Under hypoxia, both NOX and HIF-1α boost the expression of each other [59]. The stabilization of HIF-1α induces the expression of Nox1 and Nox2, generating superoxide radicals, which promotes ROS production [60]. Yuan et al. reported that hypoxia increases the mRNA expression of Nox, especially Nox2, in the brain cortex and stem of wild-type mice [61]. Additionally, the suppression of Nox2 inhibits the activation of HIF-1 through intermittent hypoxia (IH), indicating that Nox2 plays an important role in HIF-1 activation. Nevertheless, this mechanism has not been explained yet [62]. Deep et al. reported that NOX expression is crucial in PCa progression in TRansgenic Adenocarcinoma Mouse Prostate (TRAMP) mice. Additionally, Graviola pulp extract (GPE) could inhibit the hypoxia-induced NOX activity in PCa cells and decrease the expression of NOX1, NOX2, and p47phox. GPE could strongly reduce the HIF-1α levels and cause a decrease in the proliferation of PCa cells [63]. Briefly, there is a strong correlation between the expression of both HIF-1α and NOX, and this could serve as a potential chemoprevention target in controlling prostate cancer under hypoxic conditions.

#### Wnt/β-Catenin signaling pathway

**Fig.3:**
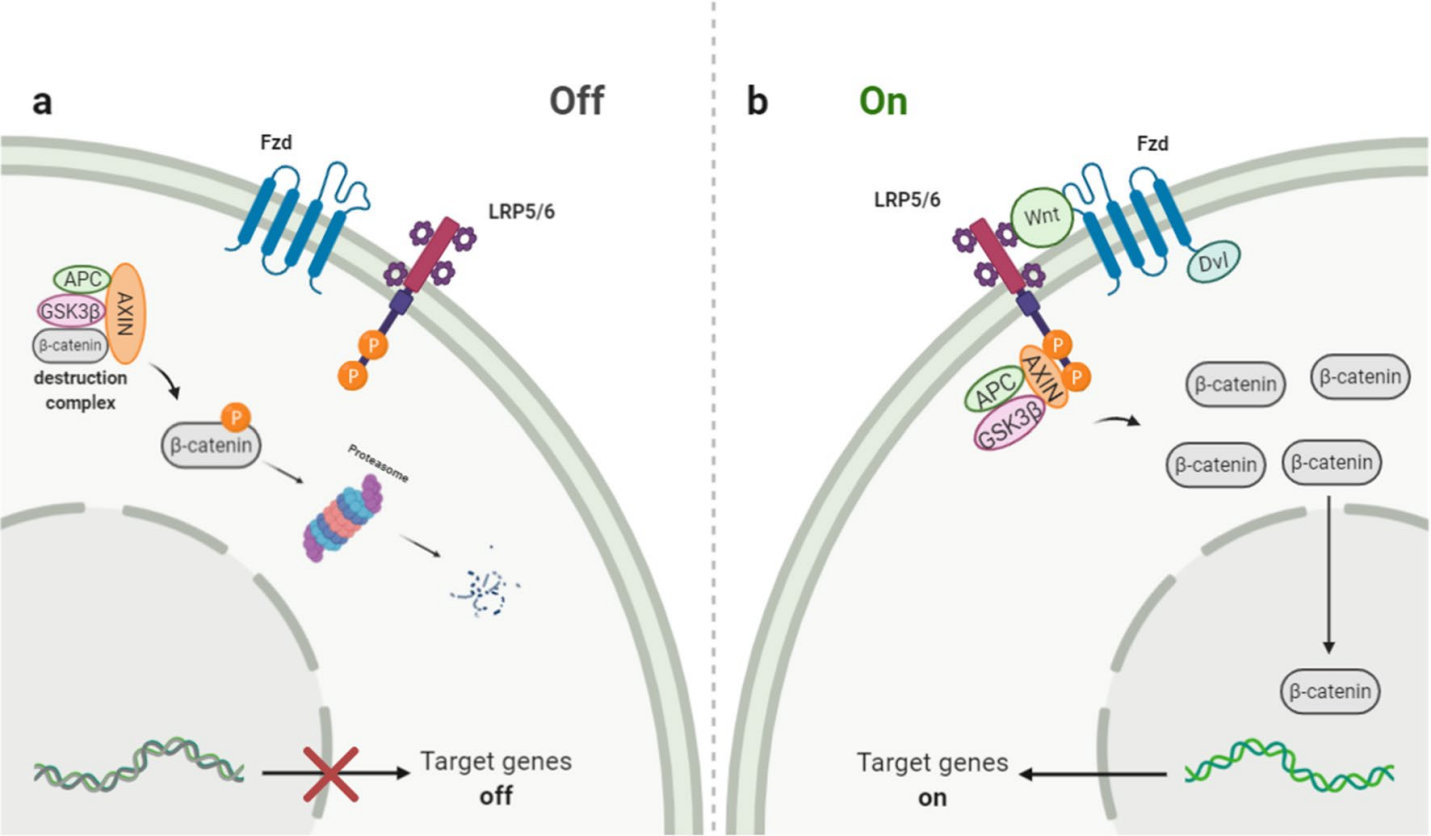
Wnt/β-Catenin Signaling pathway: The Wnt pathway is activated by the interaction of Wnt ligands with the Frizzled (Fzd) receptor and the LRP-5, 6 coreceptors. This interaction leads to the activation of the Dishevelled protein and inhibits the formation of the destruction complex. This leads to the stabilization of β-catenin, which contributes to its accumulation in the cytosol and translocation into the nucleus. In the nucleus, β-catenin activates the TCF/LEF family’s transcription factors, regulating gene expression

The Wnt/β-catenin signaling pathway, shown in Fig. 3, regulates various biological processes such as embryonic development, proliferation, differentiation, and stem cell migration. Additionally, it participates in carcinogenesis and the development of malignant neoplasms [64, 65]. In the absence of Wnt proteins, the level of β-catenin in the cytoplasm is kept very low due to the action of a complex of proteins (destruction complex) that phosphorylate it and thereby prepare for ubiquitination and subsequent degradation in the proteasomes. This complex includes two regulatory protein kinases, glycogen synthase kinase 3b (GSK-3β) and casein kinase 1α (CK1α), and β-catenin binding proteins Axin1 or Axin2 and intestinal adenomatous polyposis protein APC (adenomatous polyposis coli) [66].

The Wnt pathway is activated by the interaction of Wnt ligands (19 is known in mammals) with the Frizzled (Fzd) receptor (10 is known in mammals) and the LRP-5, six coreceptors. This interaction leads to the activation of the Dishevelled protein and inhibits the formation of the destruction complex. This leads to the stabilization of β-catenin, which contributes to its accumulation in the cytosol and translocation into the nucleus. In the nucleus, β-catenin activates the transcription factors of the TCF/LEF family (T cell factor/lymphoid enhancing factor) [66, 67].

In prostate cancer, β-catenin expression in PCa is higher than in normal prostate tissue, indicating its association with PCa progression [68]. Under hypoxic conditions, β-catenin was found to have a significant role in the progression of many cancers, including prostate and colorectal cancer [68, 69]. According to a study by Mitani et al., β-catenin and HIF-1α are associated with PCa progression through androgen receptor activation under low androgen conditions. This occurs via the binding of HIF-1α to β-catenin leading to the stimulation of its nuclear translocation and the promotion of AR transactivation [68]. Another study was carried out by To et al. on the number of human colorectal cancer cell lines. It found that hypoxia-activated the co-regulation of β-catenin and Nur77. PI3K/Akt stimulated this in a Nur77-dependent manner and independent of classical APC and p53 pathways. Also, the increased expression of both β-catenin and Nur77 under hypoxia stimulates cell migration, invasion, and epithelial-mesenchymal transition [69].

Furthermore, the Wnt/β-catenin pathway could be blocked under hypoxic conditions by activating endoplasmic stress in cancer cells. However, the Wnt/β-catenin pathway, which has common mutations, is not sensitive to this blocking [70]. In brief, the Wnt/β-catenin pathway has a significant role in the progression, metastasis, and EMT of PCa and could serve as a potential target for therapy under hypoxic conditions.

#### Hedgehog Signaling pathway

**Fig.4:**
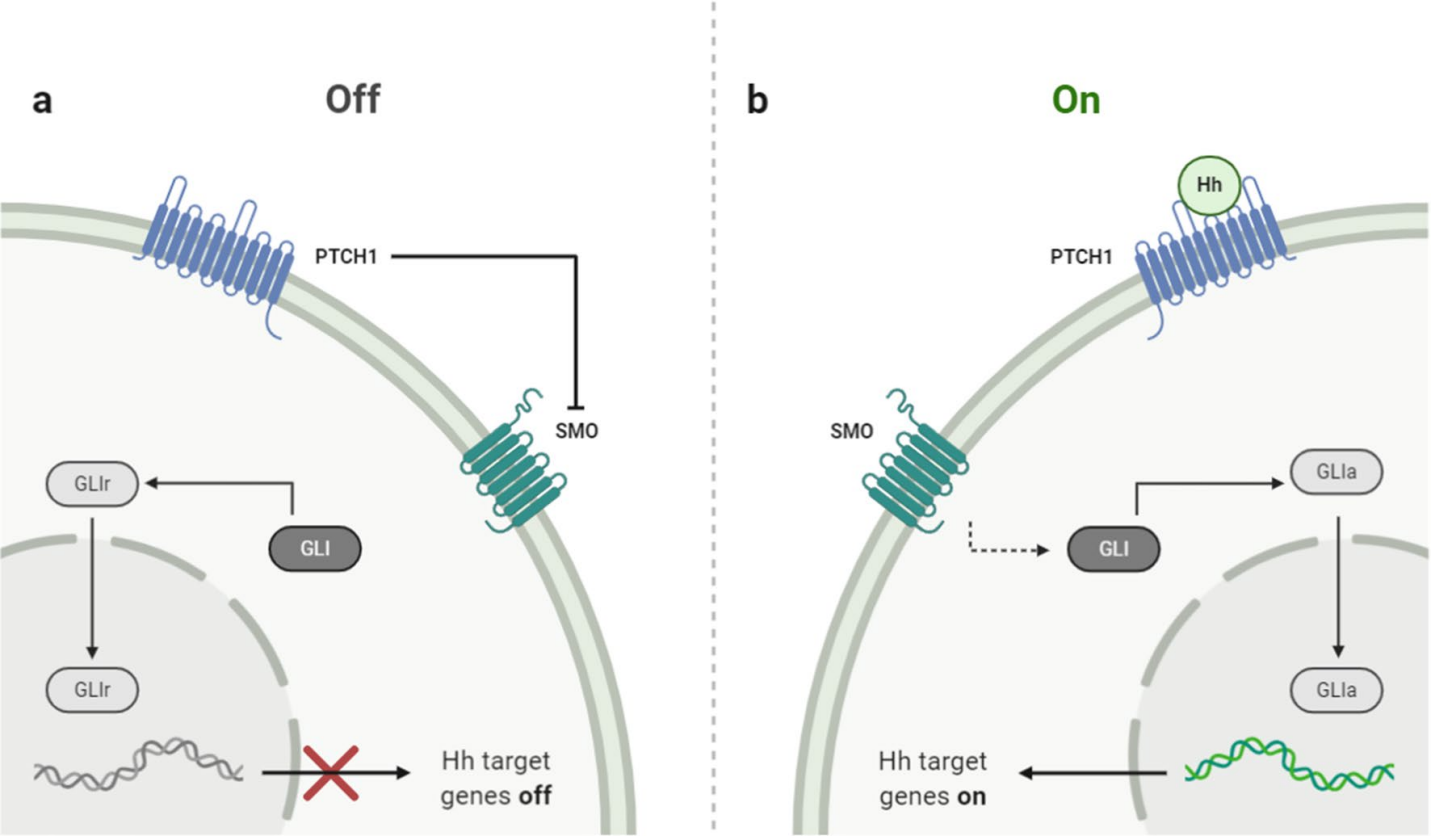
Hedgehog Signaling pathway: In the presence of Hh ligands, it binds to PTCH1. Upon this binding, the SMO's inhibitory block is removed, which activates a group of GLI proteins (the Hh signaling pathway transcription factors). In response to activation of the Hh signal, GLI proteins are differentially phosphorylated and rebuilt into transcriptional activators that activate the expression of Hh target genes by direct interaction with specific regions in the promoter region

The Hedgehog (Hh) signaling pathway, shown in Fig. 4, is requisite for normal embryonic development and plays a significant role in the maintenance and regeneration of adult tissue. The secreted Hh proteins trigger various cellular responses, ranging from cell survival and proliferation to cell fate and differentiation. In addition to its important role in normal embryonic development and adult tissue homeostasis, aberrant activation of the Hh signaling pathway is involved in several stages of carcinogenesis of different tumors, such as pancreatic, lung, prostate, ovarian, and breast cancers [71]. The key transducer of this signaling pathway is the transmembrane protein Smoothened (SMO). Its activity is inhibited by another transmembrane protein, the canonical receptor Patched (PTCH1), a cell receptor for Hh ligands. The initiation of signaling is carried out when Hh proteins bind to PTCH1. Upon this binding, the inhibitory block of SMO is removed, and as a result, the latter activates a group of GLI proteins (the Hh signaling pathway transcription factors). In response to activation of the Hh signal, GLI proteins are differentially phosphorylated and rebuilt into transcriptional activators that activate the expression of Hh target genes by direct interaction with specific regions in the promoter region. The main target genes of this signaling pathway are the glioma-associated oncogenes GLI1 and GLI2, as well as the PTCH1, PTCH2, and Hhip1 genes [71, 72]. The activation of these genes determines the number of cellular reactions, including the activation of proliferation, and anti-apoptosis of the cells [73].

Generally, there is a linkage between Hh signaling and HIF, contributing to tumor metastasis and radiation resistance. Hypoxia can activate Hh signaling pathway in both ligand-dependent and ligand-independent manner through upregulation of SMO transcription [74]. A study by Wilkinson et al. indicated that human prostate cancer-associated fibroblasts (CAFs) could form primary cilia which are related to the upregulation of canonical Hh signaling. According to our knowledge, the link between Hh signaling pathway and hypoxia has not been investigated in human PCa specimens indicating that both Hh and HIF might act independently. However, Onishi et al. reported that hypoxia stimulates the Hh signaling in pancreatic cancer samples in a ligand-independent method through the upregulation of SMO transcription. In addition, the immunocytochemical staining in human pancreatic cancer specimens detected a considerable linkage between the expression of both SMO and Gli1 and the hypoxia marker CA9 [74, 75]. This summarizes the significant role of the Hh signaling in the progression of PCa, but it has not been indicated in hypoxic PCa conditions.

#### Hypoxia-dependent EMT in prostate cancer

Epithelial-mesenchymal transition (EMT) is a morphological change in the epithelial cells, where it loses its polarity and architecture to mesenchymal cell shape [76]. Cells shift from a classical round epithelial shape with tight junctions to spindle-like mesenchymal with loose connections. It occurs during embryonic life for tissue differentiation, in adult life for tissue repair, and in cancer cells for invasion, migration, and metastasis [77, 78]. Mesenchymal cells are motile and able to invade the extracellular matrix making a path to metastasize other organs [79, 80].

Cells adapt to the hypoxic environment by increasing hypoxia-inducible factors (HIFs) expression. Mingchunli et al. proved that HIFα is responsible for EMT in prostate cancer [81, 82]. HIFα increases in short- and long-term hypoxia-treated cells. Cancer cells show less metastasis and invasion with silencing of the HIFα gene [83].

HIF alpha binds to hypoxia-responsive element (HRE) on promotors of many genes. This is how it regulates the expression of proteins and starts the EMT process. It manages the expression of cytoskeleton proteins and changes the dynamic abilities of the cells. Valenteina et al. reported an increase in annexin (ANXA1) protein expression within short-term exposure to hypoxia. Silencing ANXA 1 gene decreases the motility and invasion ability of cancer cells. ANXA1 protein upregulation is followed by losing the epithelial cell marker E-cadherin and downregulation of epithelial cytokeratin 8 and 18 (CK8, CK18). These changes result in a loss of cell-to-cell adhesion and epithelial cell architecture [82]. Expression of HIF 1 alpha and ANXA 1 protein activates the ERM family (ezrin—Radixin—measin). ERM family proteins bind actin filaments with cell membrane establishing cortical actin cytoskeleton which is required for cell migration and motility. Intermediate Filament proteins (IFs) 22 and Mitogen-Activated Protein Kinases (MAPKs) are also increased [84, 85]. Cancer invasion requires making a path in ECM, so HIFs increase the expression of proteolytic enzymes like cathepsin D, urokinase-type plasminogen activator receptor (uPAR), and matrix metalloproteinase-2 (MMP2) and factors stimulating migration like phosphoglucose isomerase/autocrine-motility factor (PGI/AMF), transforming growth factor-α (TGF-α) and the spreading factor c-Met. McMahon et al. found that the transforming growth factor beta (TGF β) signaling pathway is activated under a hypoxic microenvironment and reduces the expression of PHD enzyme resulting in the accumulation of HIFs [86].

HIF-1 binds to HRE on promotors of transcription factors Snail (Snail1, Snail2/Slug), ZEB, and basic-helix-loop-helix (Twist). Accumulating these factors in hypoxic conditions enhances EMT, as they bind to the enhancer box (E box element). Yang et al. noticed a shift from N-cadherin and vimentin markers to E-cadherin after inhibiting them.

HIF alpha stabilization and accumulation depend on AKT/mTOR pathway. Increased production of matrix metalloases and decreased expression of E-cadherin are associated with the activation of the AKT pathway. SNAIL transcription factors increase in P-AKT and target E- cadherin promotor. PI3KAKT activates RhoA and Rac 1, G protein binding proteins that regulate cytoskeleton filaments. Activating Rho and Rac enhances tumor motility and invasiveness.

Expression levels of CX3C chemokine receptor 1 (CX3CR1) are upregulated in hypoxia-treated cells at both mRNA and protein levels. HIF -1 and NF-κB pathways are responsible for this upregulation, as Li-Jie et al. noticed the downregulation of CX3CR1 after inhibiting HIF-1 and NF-κB. Furthermore, Jiebing et al. noticed a morphological change in hypoxic cells after stimulation of CX3CL1. Increased vimentin and decreased E. cadherin expression in these cells prove that CX3CL1 plays a role in EMT. CX3CL1 binds specifically to CX3CR1, followed by EGFR transactivation and slug expression. EGFR has a role in cells, differentiation, and metastasis. TGF-α is one of the ligands of EGFR, and it is essential for CX3CL1-induced EGFR transactivation. Jiebing et al. found that inhibition of TGF-α suppresses CX3CL1-induced EGFR transactivation as CXCL1 increases soluble TGF-α. TACE/ADAM17 is one of the MMP family members that is upregulated in PCa and cleaved TGF-α, so the pathway TACE/TGF-α/EGFR is the mechanism of CXCL1-induced EMT.

Hypoxia and activated androgen signaling induce monoamine oxidase A (MAOA) in PCa. The elevated expression of MAOA is followed by a decrease in E-cadherin expression and an increase in vimentin expression. Fei Liu et al. found that MAOA is elevated in PCa and drives EMT. Monoamine oxidase A (MAOA) is a mitochondrial enzyme that, when it catalyzes oxidative reactions in the outer membrane of mitochondria, immediately produces hydrogen peroxide as a by-product that can be further converted into other species of ROs. Accumulation of ROS augments hypoxic conditions and stabilizes HIF alpha, as it inhibits PHD without a change in HIF alpha transcription [87]. Elevated ROS stabilizes HIF alpha which induces mitochondrial activity to produce ROS. This vicious cycle worsens the hypoxic conditions and enhances prostate cancer tumorigenesis and metastasis. Elevated MAOA signaling also increases the expression of vascular endothelial growth factor A (VEGF-A) and its coreceptor NRP1, facilitating PCa metastasis to the bone and vertebral column. MAOA increases the expression of TWIST through the effect of HIF alpha and even after HIF alpha knockdown. MAOA/AKT/FOXO 1 signaling directly binds to response elements at the TWIST promotor [86, 88].

#### Hypoxia and androgens

Androgens are male sex steroid hormones such as testosterone and its more potent metabolite, 5α-dihydrotestosterone (DHT) [89]. The androgen receptor (AR) performs a ligand-dependent transcriptional factor that belongs to the steroid hormone receptor superfamily. AR is pivotal in developing androgen-dependent and castration-resistant prostate cancer (CRPC) [90]. In the cytoplasm present, the ligand-free AR is in an inactive form which consists of an NH2-terminal unstructured transcriptional activation domain (NTD), the central DNA binding domain (DBD), and the carboxyl-terminal ligand binding domain (LBD) [91]. Androgens bind AR (in the LBD), causing its activation. This active form plays an important role in the development and differentiation of prostate cancer cells [92]. The conformational change occurs by the N–C interaction (interaction between the NH2-terminal and COOH-terminal regions of AR), which is enhanced by coactivators such as SRC-1, ARA24, and β-catenin [93]. The ligand-bound AR can translocate into the nucleus and bind to androgen response elements (AREs) in the promoter regions such as NKX3*.1* and PMEPA-1. This binding is enhanced by transcriptional coactivators such as p160 proteins, ARA proteins GAPDH, and β-catenin [90]. Hypoxia enhances the binding of AR to AREs in the promoter regions of NKX3.1 and PMEPA-1 genes[94]. Further, HIF-1α enhanced the β-catenin, which activated the N–C interaction of AR and developed the physical interaction between NTD and LBD of AR [93]. Also, hypoxia stimulates the phosphorylation levels of p38 mitogen-activated protein kinase (MAPK) and HSP27, which bind with AR, causing enhancement of AR stability, shuttling, and transcriptional activity [94]. Therefore, the most effective treatment for prostate cancer is androgen deprivation therapy (ADT). The ADT depends on the repression of AR signaling by blocking the production of androgens or by repressing the androgen binding to the AR, which can achieve a response of over 80% [95]. Metastatic CRPC is caused by converting androstenedione to 5a-androstenedione, which is converted to dihydrotestosterone, bypassing testosterone [91].

Studies indicated that prostate cancer bone metastases could convert adrenal androgens to testosterone and dihydrotestosterone. The risk of CRPC increases ten times in hypoxic cells and tumors expressing HIF1a. Therefore, inhibiting hypoxia may be vital in increasing the efficacy of AR-targeted therapy [95].

#### Hypoxia as a potential target therapy

Hypoxia is a common feature of solid tumors and contributes to their aggressive phenotype, metastasis, and resistance to therapy. Emerging therapeutic strategies, such as inhibiting HIF-1α signaling or hypoxia-activated prodrugs (HAPs), provide new insights to overcome the hypoxia barrier. For targeting hypoxia signaling pathways, it has been found that the Wnt signaling correlates with drug resistance in prostate cancer. The combination of β-catenin inhibitor ICG001 with enzalutamide, which is an anti-cancer hormonal therapy, overcomes enzalutamide resistance and restores its sensitivity in CRPC [96]. Also, suppression of the noncanonical Wnt pathway overcomes enzalutamide resistance in CRPC. Regarding the PI3K/Akt/mTOR pathway, the YC-1 drug has been found to inactivate this pathway by reducing the accumulation of HIF-1a and HIF-1b and antagonizing the activation of nuclear factor (NF-kB) induced by hypoxia in prostate cancer cells [97].

Another approach for targeting hypoxia is hypoxia-activated prodrugs (HAPs), which are selectively activated in low-oxygen environments by cellular reductases. They can target HIF mRNA and protein expression, dimerization, DNA binding, and transcriptional activity of HIFs. HAPs such as AQ4N combined with single-dose radiation result in a marked increase in antitumor efficacy without any enhancement of functional loss compared to radiation alone in mice-bearing tumors [98]. Also, OCT1002, an analogue of AQ4N, has a hypoxia-dependent anti-tumor effect when combined with bicalutamide, and inhibits ADT resistance and progression to CRPC in LNCaP prostate tumor xenografts [99]. Despite promising results from preclinical studies, many HAPs have a dissatisfying performance in clinical trials.

Finally, a stronger focus on how these hypoxia-related pathways work will further help in designing new therapeutic approaches to overcome the obstacles associated with therapy resistance.

## Conclusion

Hypoxia regulates the prostatic tumor microenvironment, stromal component, and extracellular matrix (ECM). Under hypoxic conditions, the HIF signaling pathway is activated. This major signaling pathway promotes adaptive tumor processes and helps in the activation of other hypoxia-induced pathways, including PI3K/Akt/mTOR, NOX, Wnt/β-Catenin, and Hedgehog signaling pathways promoting tumor survival. In addition, hypoxia promotes epithelial-mesenchymal transition (EMT) recurrence and metastasis. Moreover, hypoxic conditions enhance androgen receptor (AR) stability, shuttling, and transcriptional activity that contributes to the development of androgen-dependent cancer and castration-resistant prostate cancer (CRPC). Therefore, a better understanding of the hypoxic microenvironment and hypoxia-induced pathways will lead to the discovery of promising novel therapeutic targets and the overcoming of drug resistance issues.

## Supplementary Information

Below is the link to the electronic supplementary material.

Supplementary file1 (TIFF 2405 KB)
