# Supplementary figures and images for: The role of hypoxia on prostate cancer progression and metastasis
Source: Mol Biol Rep. 2023 Feb 14;50(4):3873–84. doi: 10.1007/s11033-023-08251-5 (PMC10042974; doi:10.1007/s11033-023-08251-5)

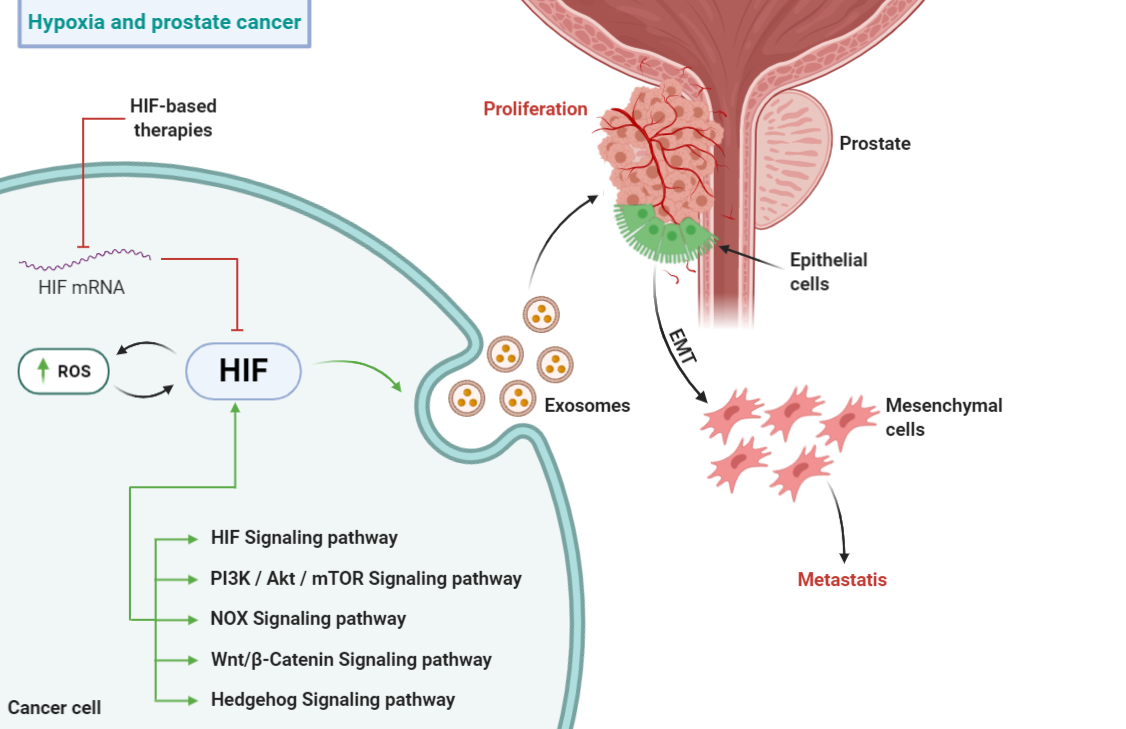

Supplement: Supplementary file 1 — Supplementary file1 (TIFF 2405 KB) [file 11033_2023_8251_MOESM1_ESM.tiff]
